# Alternative culture medium design for biomass production of autochthonous meat starter *Latilactobacillus sakei* sp. *sakei* ACU-2

**DOI:** 10.1038/s41598-023-29502-2

**Published:** 2023-03-30

**Authors:** Nadia Galante, Noelia Palavecino Prpich, Carmen Campos, María Elisa Cayré, Marcela Castro

**Affiliations:** 1 Laboratorio de Microbiología de Alimentos, Instituto de Investigaciones en Procesos Tecnológicos Avanzados (INIPTA), Consejo Nacional de Investigaciones Científicas y Técnicas (CONICET), Universidad Nacional del Chaco Austral (UNCAus), Comandante Fernández 755, (3700) Sáenz Peña, Chaco Argentina; 2 Departamento de Industrias, Instituto de Tecnología de Alimentos y Procesos Químicos (ITAPROQ), Consejo Nacional de Investigaciones Científicas y Técnicas (CONICET), Av. Int. Güiraldes s/n, (1428) Ciudad Autónoma de Buenos Aires, Argentina

**Keywords:** Applied microbiology, Biotechnology

## Abstract

The autochthonous strain *Latilactobacillus sakei* sp. *sakei* ACU-2 was selected as a meat starter culture for dry sausage production. Transferring this strain from laboratory scale to industry requires an increase in biomass production, while lowering process costs. In this study, a combination of techniques was applied in order to optimize the culture medium composition to enhance biomass production of *L. sakei* ACU-2. One variable at a time experiments, Plackett–Burman design, and mixture design were performed to fulfill the strain nutritional requirements. Eventually, the optimized formulation contained 19.46 g/L yeast extract; 8.28 g/L whey protein concentrate; 2.26 g/L soy peptone; 30 g/L cerelose; 1 g/L Tween 80; 5 g/L sodium acetate; 0.2 g/L magnesium sulfate and 0.05 g/L manganese sulfate. When *L. sakei* ACU-2 was cultivated in a bioreactor using the alternative medium, an enhancement of 75.5% of biomass production was achieved, in comparison to its growth in the commercial de Man, Rogosa, and Sharpe medium. Furthermore, a reduction of 62–86% of the cost was also attained. These results support a promising large-scale application of the designed medium for high biomass yields of the starter culture at minor costs.

## Introduction

Starter cultures comprise beneficial bacteria that are vital to the production of dry sausages, increasing food safety and guaranteeing uniformity in the production process and stability throughout shelf life. Although artisanal fermented products are conceived by spontaneous fermentation from the in-house flora of the manufacturing plant, introduction of especially designed starter cultures into the production line brings the before mentioned benefits together with the maintenance of valuable organoleptic characteristics. Keeping the original flavors is a must that can be achieved by means of autochthonous bacteria isolated from the product. In this sense, *Lactobacillus sakei* ACU-2, recently renamed as *Latilactobacillus sakei* sp. *sakei* ACU-2^1^ was isolated from fermented meat products and tested as a starter culture in a small-scale facility^2^. The strain showed a good performance in the manufacturing of dry fermented sausages, leading the fermentation process and improving food safety and quality, without compromising the identity of the final products. Thereby, the addition of this culture to the production line is a potentially useful tool to achieve a competitive meat fermented product at regional markets.

The transfer of *L. sakei* ACU-2 from laboratory scale to industry requires an increase in biomass production, while lowering process costs. The de Man, Rogosa and Sharpe (MRS) culture medium is frequently used for lactobacilli growth, however, its high price and low yields in the overproduction of some strains make it inadequate for industrial purposes^3^. Low-cost alternative use of dairy by-products and agro-industrial wastes has gained attention in the last years, mainly as substitutes of carbon and nitrogen sources in culture media. Corn steep liquor, maltose syrup, cane molasses, wheat germ extract, soy hydrolysates and cheese whey, and their proteinaceous concentrates, are the most commonly agro-residues used for lactic acid bacteria (LAB) cultivation^4–8^. Being complex sources of micro and macro nutrients (chiefly high concentrations of carbon and nitrogen), these residues are appropriate as fermentable substrates for nutritional demanding microorganisms, such as LAB. However, biomass yields obtained with several complex sources display batch-to-batch variations^3^. Consequently, a correct balance between chemical defined and complex substrates must be established, in order to develop an efficient fermentation medium for industrial scale.

The design of alternative culture mediums is mainly influenced by nutritional requirements and physiological properties of microorganisms. Therefore, medium composition and growth conditions should be optimized according to the target strain^9^. Optimization is a necessary step in biotechnological processes in order to maximize yields and minimize production costs^10^. Usually, a sequence of several techniques is applied, combining classical and statistical optimization methods. Among the classical approaches, the one variable at a time (OVAT) methodology is used as a preliminary stage in the study of medium components and physicochemical parameters. Even though this procedure is laborious and time consuming, it helps to define strain basic requirements and establish independent variables for the subsequent experiments^11^.

Statistical techniques, such as the design of experiments (DOE) and response surface methodology (RSM), comprise the set-up of carefully planned assays to statistically evaluate the effect of the variables on the response and attain optimization^11^. In DOE, the Placket Burman design (PBD) aids with the screening and selection of contributing factors. This design has been successfully executed in the identification of the most influential medium components for metabolite production and growth promotion of several LAB^12–16^. Despite its efficiency in defining main effects, PBD ignores interactions among the variables; which necessarily leads to a next strategy.

Mixture designs are a type of RSM used to study the interactions between independent variables (proportions of the components) and their effect on the response, when the response depends only on the proportion of the mixture components and such proportions are not independent between them^17^. This type of experiment is usually employed in product development whenever a multicomponent system is involved in order to achieve a specified quality and suitable technological properties. Among the designs for mixtures, simplex lattice and simplex centroid designs are the most widely used. Simplex centroid designs are reported to be model independent and require fewer number of experiments than simplex lattice designs^18^.

Scaling up procedure—involving technological transfer—implies the concurrence of many factors that play substantial roles in biomass production. The orchestration of these actors demands a careful and systematic study. In this research a combination of optimization techniques was applied in order to design an inexpensive food-grade fermentation medium to enhance biomass production of *L. sakei* ACU-2.

## Materials and methods

### Microorganism and culture conditions

*Latilactobacillus sakei* sp. *sakei* ACU-2, isolated from artisanal dry fermented sausages manufactured in Chaco province (Argentina), is one of the strains that comprise an autochthonous meat starter culture^19^. The strain is deposited in the strain repository of Laboratorio de Microbiología de Alimentos (INIPTA-CONICET-UNCAus) and kept in MRS broth supplemented with 20% (v/v) glycerol at a temperature of − 80 °C. After 12 h incubation at 30 °C, the culture suspension was centrifuged (DragonLab D3024R, China) at 10,000 rpm (10 min, 4 °C); the obtained pellet was washed and resuspended in an appropriate volume of 0.85% (w/v) NaCl solution to obtain an optical density (OD_600_) of 1.8 ± 0.2 (~ 9 log CFU/mL).

### Hydrolysis of whey protein concentrate (WPC)

The hydrolysis of WPC was carried out prior to each assay in order to enhance protein properties^20^. Following the technique described in Manzoor et al.^5^, WPC was reconstituted in distilled water to obtain a solution with the required concentration for each trial, as follows: 25, 12.5 and 5 g/L in OVAT experiments; 2, 6 and 10 g/L in PBD; 30, 15 and 10 g/L in mixture design; and 8.28 g/L in the bioreactor cultivation study. This solution was subjected to acidic hydrolysis with 10 M HCl until reaching a pH of 4.0 and subsequent heating at 100 °C for 10 min. The obtained hydrolysate was cooled to room temperature and then filtered to remove solids.

### Biomass determination

Samples consisting of 10 ml of culture broth were collected after 24 h of fermentation and centrifuged (Presvac DCS-16-RV, Argentina) at 8,000 rpm for 15 min at room temperature. Supernatants were discarded and the pellets were washed twice with 0.85% (w/v) NaCl solution. Finally, dry cell mass was determined by drying at 80 °C until it reached constant weight.

### Optimization of culture medium formulation

A sequence of optimization techniques was applied to obtain a suitable fermentation medium to enhance the growth of *L. sakei* ACU-2. These experiments were conducted in 100 ml flasks containing 50 ml of culture medium inoculated at 1% (v/v). After 24 h of fermentation, biomass production was assessed by the technique described in "Biomass determination" section.

#### One variable at a time experiments

The OVAT method was used as a preliminary study to define the nutrients and physicochemical parameters which most influenced biomass production of *L. sakei* ACU-2.

When physicochemical variables were evaluated, commercial MRS broth (Biokar Diagnostics, France) was used as fermentation medium. The effects of initial pH (4.5, 5.5, 6.5 and 7.5) were assayed under a controlled temperature of 30 °C and the influence of different fermentation temperatures (25, 30, 37 and 42 °C) was evaluated with an initial pH of 6.5 ± 0.2.

**Figure 1:**
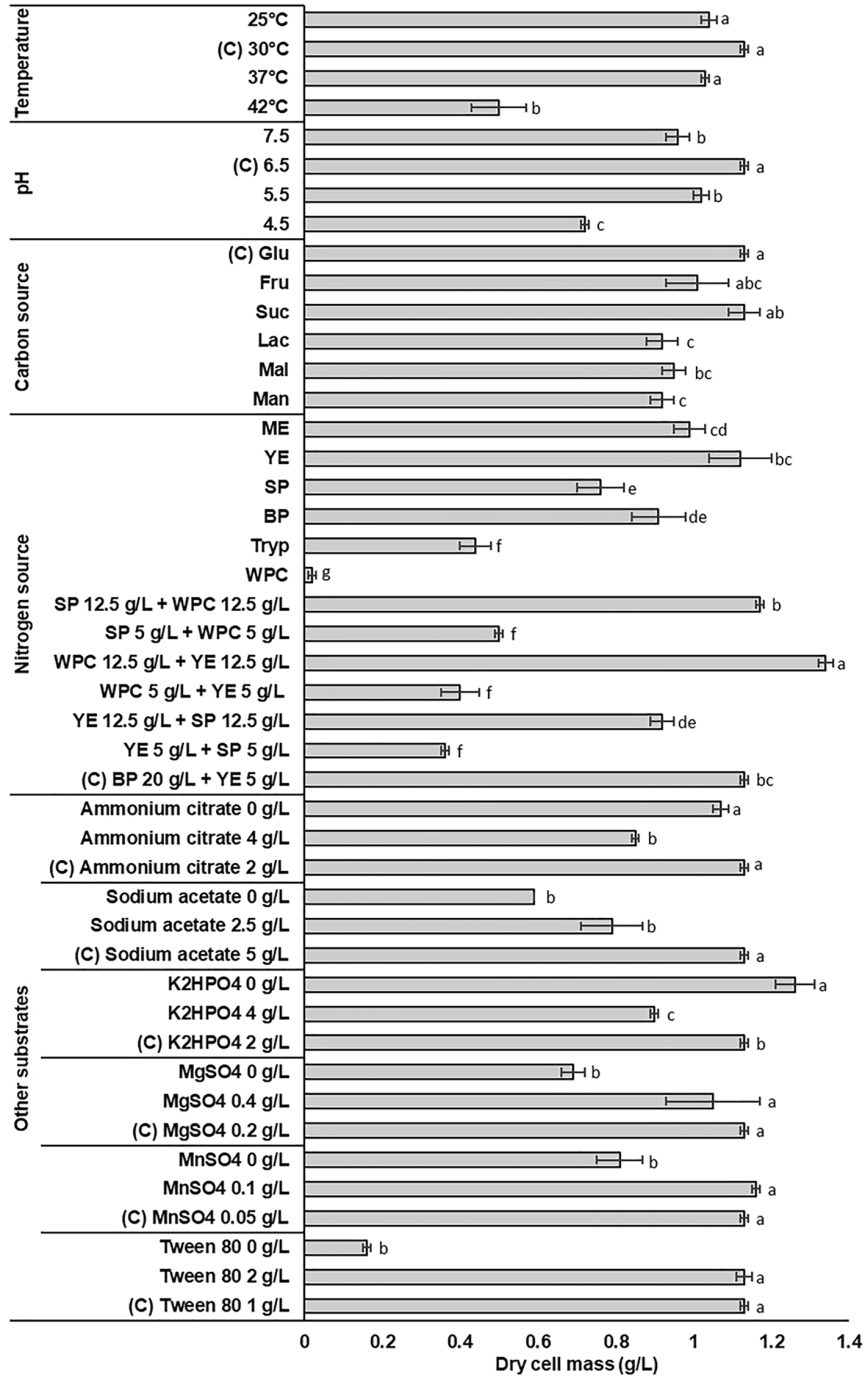
Effects of different incubation temperatures, initial pH, carbon sources, organic nitrogen sources, salts and Tween 80 on the biomass production of *L. sakei* ACU-2. Results are the mean values of the triplicates and the error bars indicate the standard deviation. Different letters within groups represent significant differences (*p* < 0.05, Tukey test). (C) Control.

To determine the most influential carbon and nitrogen sources on bacterial cell mass, a basal medium consisting of MRS broth formulation (20 g/L glucose, 20 g/L beef peptone, 5 g/L yeast extract, 5 g/L sodium acetate, 2 g/L ammonium citrate, 2 g/L K_2_HPO_4_, 0.2 g/L MgSO_4_, 0.05 g/L MnSO_4_, 1.08 g/L Tween 80) was used. The selected carbohydrates were glucose, sucrose, lactose, fructose, maltose and mannose, and the organic nitrogenous compounds consisted of meat extract (ME), yeast extract (YE), soy peptone (SP), beef peptone (BP), tryptone and WPC. Each of them was added individually to the basal medium at a concentration of 20 and 25 g/L for carbon and nitrogen sources, respectively. Binary combinations of three nitrogen compounds (WPC-YE, YE-SP, WPC-SP) were also tested in different concentrations, as it can be seen in Fig. 1. In addition, the influence of different concentrations (g/L) of sodium acetate (0, 2.5, 5), ammonium citrate (0, 2, 4), K_2_HPO_4_ (0, 2, 4), MgSO_4_ (0, 0.2, 0.4), MnSO_4_ (0, 0.05, 1) and Tween 80 (0, 1, 2) were screened. All the experiments were performed at 30 °C and initial pH of 6.5 ± 0.2.

All chemicals and carbohydrates were purchased from either Sigma-Aldrich (USA) or Cicarelli (Argentina). Peptones and extracts were acquired from Britania (Argentina) and WPC (Lacprodan 80) from Arla Food Ingredients S.A. (Argentina).

#### Plackett–Burman design

The PBD was applied to identify the most significant variables that affected biomass production. Based on OVAT results, a mixture of growth factors at a fixed concentration of 5.25 g/L (5 g/L sodium acetate, 0.2 g/L MgSO_4_ and 0.05 g/L MnSO_4_) and 1 g/L of Tween 80 was chosen for a new and simpler basal medium, and a total of six medium components including three carbon sources (glucose, sucrose and fructose) and three nitrogen sources (WPC, yeast extract and soy peptone) were selected for PBD. In order to reduce the cost of the medium, glucose was replaced by an industrial and economical alternative, cerelose (Ingredion, Argentina) and both sucrose and fructose were of commercial origin. Each component was tested at low (− 1) and high (+ 1) concentration levels in 12 runs, plus four central points for a total of 16 trials, as shown in Table 1. Initial pH value and incubation temperature parameters were also adjusted to the results of OVAT experiments. The following first-order polynomial equation was used to perform mathematical modeling:

1 $$Y = \beta_{0} + \sum \beta_{i} X_{i}$$

where *Y* is the response, *β*_0_ is the model intercept, *β*_i_ the regression coefficients and *X*_i_ represents the independent variable. Variables with significant effect on biomass production were considered for further optimization.

**Table 1:** Experimental matrix of the Plackett–Burman design for biomass production of *L. sakei* ACU-2.

Run	*X*_1_	*X*_2_	*X*_3_	*X*_4_	*X*_5_	*X*_6_	Dry cell mass (g/L)	
							Experimental values	Predicted values
1	+ 1(10)	− 1 (2)	+ 1 (10)	− 1 (10)	− 1 (10)	− 1 (10)	1.41 ± 0.02	1.38
2	+ 1 (10)	+ 1 (10)	− 1 (5)	+ 1 (30)	− 1 (10)	− 1 (10)	1.53 ± 0.05	1.60
3	− 1 (5)	+ 1 (10)	+ 1 (10)	− 1 (10)	+ 1 (30)	− 1 (10)	1.39 ± 0.03	1.40
4	+ 1 (10)	− 1 (2)	+ 1 (10)	+ 1 (30)	− 1 (10)	+ 1 (30)	1.38 ± 0.01	1.38
5	+ 1 (10)	+ 1 (10)	− 1 (5)	+ 1 (30)	+ 1 (30)	− 1 (10)	1.70 ± 0.12	1.60
6	+ 1 (10)	+ 1 (10)	+ 1 (10)	− 1 (10)	+ 1 (30)	+ 1 (30)	1.71 ± 0.01	1.71
7	− 1 (5)	+ 1 (10)	+ 1 (10)	+ 1 (30)	− 1 (10)	+ 1 (30)	1.43 ± 0.04	1.40
8	− 1 (5)	− 1 (2)	+ 1 (10)	+ 1 (30)	+ 1 (30)	− 1 (10)	1.05 ± 0.06	1.07
9	− 1 (5)	− 1 (2)	− 1 (5)	+ 1 (30)	+ 1 (30)	+ 1 (30)	0.96 ± 0.04	0.96
10	+ 1 (10)	− 1 (2)	− 1 (5)	− 1 (10)	+ 1 (30)	− 1 (10)	1.24 ± 0.07	1.27
11	− 1 (5)	+ 1 (10)	− 1 (5)	− 1 (10)	− 1 (10)	+ 1 (30)	1.28 ± 0.04	1.29
12	− 1 (5)	− 1 (2)	− 1 (5)	− 1 (10)	− 1 (10)	− 1 (10)	1.02 ± 0.03	0.96
13	0 (7,5)	0 (6)	0 (7,5)	0 (20)	0 (20)	0 (20)	1.31 ± 0.04	1.33
14	0 (7,5)	0 (6)	0 (7,5)	0 (20)	0 (20)	0 (20)	1.39 ± 0.02	1.33
15	0 (7,5)	0 (6)	0 (7,5)	0 (20)	0 (20)	0 (20)	1.27 ± 0.01	1.33
16	0 (7,5)	0 (6)	0 (7,5)	0 (20)	0 (20)	0 (20)	1.29 ± 0.07	1.33

*X*_1_ Yeast extract (g/L); *X*_2_ WPC (g/L); *X*_3_ Soy peptone (g/L); *X*_4_ Cerelose (g/L); *X*_5_ Sucrose (g/L); *X*_6_ Fructose (g/L).

(+ 1), highest concentration of variable; (− 1), lowest concentration of variable; (0), central concentration of variable.

The dry cell mass was measured after 24 h of incubation. Data are presented as the mean value of independent experiments performed in triplicate and the standard deviation.

#### Mixture design for nitrogen source selection

In order to obtain the optimum mixture composition of organic nitrogen source that promoted the growth of *L. sakei* ACU-2, a three-component simplex-centroid mixture design with constraints was applied. According to the PBD results, WPC, yeast extract and soy peptone were the components selected for the mixture while basal medium was added to a fixed concentration of 30 g/L of cerelose. The total amount of mixture was restricted at a maximum of 30 g/L and each one of the components was studied in four levels: 0 (0%), 1/3 (33%), 1/2 (50%) and 1 (100%), as shown in Table 2. The design consisted of 7 runs: three points at the extreme vertices, three points at the edge centroids and one point at the overall centroid. Each experimental run was replicated once for a total of 14 trials. Experiments were performed under random conditions and data was fitted for variations of the studied response to linear, quadratic and special cubic regression models (Eq. 2, 3 and 4, respectively).

2 $$Y = \sum \beta_{{\text{i}}} X_{{\text{i}}}$$

3 $$Y = \sum \beta_{{\text{i}}} X_{{\text{i}}} + \sum \beta_{{{\text{ij}}}} X_{{\text{i}}} X_{{\text{j}}}$$

4 $$Y = \sum \beta_{{\text{i}}} X_{{\text{i}}} + \sum \beta_{{{\text{ij}}}} X_{{\text{i}}} X_{{\text{j}}} + \sum \beta_{{{\text{ijk}}}} X_{{\text{i}}} X_{{\text{j}}} X_{{\text{k}}}$$

where *Y* is the predicted response, *β*_i_ is the regression coefficient for each linear effect term, *β*_ij_ is the binary interaction effect term, *β*_ijk_ is the interaction of three components effect term and *X*_i_, *X*_j_, *X*_k_ are the independent variables.

**Table 2:** Matrix of the simplex centroid design applied to studying the biomass production of *L. sakei* ACU-2 in the medium containing different nitrogen sources.

Run	*X*_1_	*X*_2_	*X*_3_	Dry cell mass (g/L)	
				Experimental values	Predicted values
1	1 (30)	0 (0)	0 (0)	1.41 ± 0.03	1.43
2	0 (0)	1 (30)	0 (0)	0.01 ± 0.00	0.01
3	0 (0)	0 (0)	1 (30)	1.11 ± 0.04	1.08
4	0.5 (15)	0.5 (15)	0 (0)	1.49 ± 0.04	1.59
5	0.5 (15)	0 (0)	0.5 (15)	1.49 ± 0.08	1.50
6	0 (0)	0.5 (15)	0.5 (15)	1.22 ± 0.01	1.24
7	0.33 (10)	0.33 (10)	0.33 (10)	1.73 ± 0.01	1.64
8	1 (30)	0 (0)	0 (0)	1.47 ± 0.05	1.43
9	0 (0)	1 (30)	0 (0)	0.02 ± 0.04	0.01
10	0 (0)	0 (0)	1 (30)	1.07 ± 0.11	1.08
11	0.5 (15)	0.5 (15)	0 (0)	1.63 ± 0.01	1.59
12	0.5 (15)	0 (0)	0.5 (15)	1.45 ± 0.01	1.50
13	0 (0)	0.5 (15)	0.5 (15)	1.20 ± 0.05	1.24
14	0.33 (10)	0.33 (10)	0.33 (10)	1.66 ± 0.02	1.64

*X*_1_ Yeast extract (g/L); *X*_2_ WPC (g/L); *X*_3_ Soy peptone (g/L). Where *X*_1_ + *X*_2_ + *X*_3_ = 30 g/L.

The dry cell mass was measured after 24 h of incubation. Data are presented as the mean value of independent experiments performed in triplicate and the standard deviation.

The best fitted model was selected based on the analysis of variance (ANOVA), R^2^, adj-R^2^ and pred-R^2^ values. Predictive performance of the model was validated. Five extra assays were performed and a comparison between experimental and predicted values were made within a 95% confidence interval. Accuracy factor (*AF*) and bias factor (*BF*) were assessed as described by Baugreet et al.^21^.

5 $$AF = 10^{{\frac{{\sum \log \left| {\frac{{V_{P} }}{{V_{E} }}} \right|}}{{n_{e} }}}}$$

6 $$BF = 10^{{\frac{{\sum \log \left( {\frac{{V_{P} }}{{V_{E} }}} \right)}}{{n_{e} }}}}$$

Efficiency of the data to fit the model was evaluated through average mean deviation *E* (%)^22^.

7 $$E\left( \% \right) = \frac{1}{{n_{e} }}\sum\nolimits_{i = 1}^{n} {\left| {\frac{{V_{E - } V_{P} }}{{V_{E} }}} \right|100}$$

where *n*_e_ is the number of observations and *V*_*E*_ and *V*_*P*_ are the experimental and predicted values, respectively.

### Bioreactor cultivation study

Fermentations under the optimized and commercial MRS culture medium were conducted in a 2 L reactor (Biostat A, Sartorius Stedim Biotech, Germany), with a working volume of 0.75 L for 24 h, as a means to compare growth performance of the strain. Inoculum size was 4% (v/v), while fermentation conditions were kept as following: temperature 30 °C, pH at a constant value of 6.5 (with 1.25 M NaOH and 1.25 M HCl) and agitation speed 200 rpm.

### Statistical analysis

Determinations were made by triplicate. Results are presented as the mean value ± standard deviation (SD). All designs and calculations were performed by Statgraphics plus 4.0 software (Statistical Graphics Corp., Rockville, MD, USA). The effect of the variables on biomass production was investigated through ANOVA with a confidence level of 95%. Tukey test was used to determine significant differences between the means.

## Results and discussion

### One variable at a time experiments

Incubation temperature and initial pH value, as well as main components of the culture medium, such as carbon and complex nitrogen sources, salts and surfactant, were assayed in order to define the most appropriate ones for the growth of *L. sakei* ACU-2. The results of dry cell mass (g/L) obtained varying one of the factors while the others were kept constant, are depicted in Fig. 1.

The studied strain showed the highest cell density when the initial pH of the medium was 6.5 (*p* = 0.0002), displaying a clear preference for neutral conditions. Typically, the optimal pH range for LAB is between 5.0 and 6.0, however, some authors have reported the tendency of *Lactobacillus* strains to grow better at neutral pH^7,9^.

*Latilactobacillus sakei* ACU-2 growth was also affected by the different incubation temperatures tested. While no significant differences were detected in biomass production when the incubation temperature was 25, 30 or 37 °C, this production was clearly lower at 42 °C (*p* = 0.0002). Many authors reported higher LAB biomass production with incubation temperatures ranging from 22 to 40 °C^5,9,23^. However, *L. sakei* strains have been documented to grow at temperatures between 5 and 35 °C but not higher^24^.

Figure 1 also illustrates that the presence of some salts and the absence of others had a positive effect on the development of the studied strain. Magnesium and manganese sulfates, and sodium acetate, at control concentrations (0.2, 0.05 and 5 g/L, respectively), yielded higher biomass values than their absence. On the other hand, better results were obtained when dipotassium phosphate and ammonium citrate were absent from the culture medium. Stimulatory effect of ions Mn^2+^ and Mg^2+^ on LAB growth has been well documented^25–27^ and is commonly attributed to their contribution to nutrient transportation and enzymatic activities. Following the same fashion, addition of sodium acetate, a buffering agent, growth cofactor and selective agent for lactobacilli, was reported to be necessary for culture mediums^27–29^. Contradictory results have been informed about the influence of dipotassium phosphate and ammonium citrate on the growth of LAB. In some cases, they were essential for microbial development, particularly in strains sensitive to pH decrease^7,30^, whereas in others their addition could be omitted^27,28^.

A non-ionic surfactant (Tween 80) was assayed and its presence in the medium produced higher dry cell mass values (1.13 ± 0.01) than the one in its absence (0.16 ± 0.01). Enhancement of Tween 80 and other polysorbates on lactobacilli growth has been attributed to their ability to increase fluidity of LAB membranes and to prevent cell damage due to adverse environmental conditions, such as acidity, freeze-dried and nutrient depletion^25,31,32^.

Among the six carbon sources evaluated, no considerable difference in dry cell mass values were detected between glucose, sucrose and fructose, while lactose, maltose and mannose yielded lower values. Lactic acid bacteria use a wide range of simple sugars for their growth. As regards *L. sakei* strains, glucose, sucrose and fructose have been reported to be the preferred carbohydrates for their metabolism^33^. Moreover, Lee et al.^34^ informed that glucose and fructose comprise the primary energy sources—during the initial growth phase—for *L. sakei*.

Concerning the selection of organic nitrogen sources, single compounds as well as combinations of some of them were tested. The highest biomass values were obtained when medium nitrogen source consisted of WPC combinations with food-grade substrates, such as YE (1.34 ± 0.02 g/L) or SP (1.17 ± 0.01 g/L), at a final concentration of 25 g/L. No substantial differences were detected between the latter combination and the control (1.13 ± 0.01 g/L). When WPC was tested by itself, the lowest biomass values were detected (0.02 ± 0.01 g/L); this fact suggests that its combinations might be growth promoters. Since protein hydrolysates contain a lower proportion of free amino acids and longer peptides than extracts, especially yeast extract, it is not surprising that the combination of these two sources gives better results than if used individually.

The use of hydrolysates to enhance the growth of several bacteria has caught attention in the last years and has led to varied results. Supplementation of skim milk with dairy-based proteins was studied by Prasanna et al.^35^ and the addition of casein hydrolysate resulted in a higher cell growth of *Bifidobacterium* strains when compared to WPC, whey protein isolate and lactalbumin hydrolysate. Aasen et al.^36^ compared the growth of *L. sakei* CCUG 42687 in different complex nutrients finding that fish-protein hydrolysate had minor effects on the final cell mass. On the contrary, Atilola et al.^37^ observed an increase in cell population when phytone peptone was added to the basal medium.

The most critical components of culture medium are carbon and nitrogen sources. Consequently, once the nutritional requirements for the *Lactobacillus* strain are met, optimal growth will be achieved. Hence, the carbohydrates and complex nitrogen sources that best performed in the preliminary studies conducted were selected as variables for the next design, whereas the concentrations of sodium acetate, magnesium and manganese sulfates, and Tween 80 were fixed at 5, 0.2, 0.05 and 1 g/L, respectively. The initial pH of the basal medium was defined at 6.5 and the incubation temperature was kept at 30 °C.

### Plackett Burman design

In order to define the main effects of carbon and nitrogen sources on biomass production of *L. sakei* ACU-2, a PBD was applied. Cerelose, sucrose, fructose, YE, WPC and SP were the variables selected for the design, in accordance with results of previous assays. *L. sakei* ACU-2 was able to grow in the 16 experimental runs and the values obtained for dry cell mass ranged from 0.96 ± 0.04 to 1.71 ± 0.01 g/L (Table 1).

**Figure 2:**
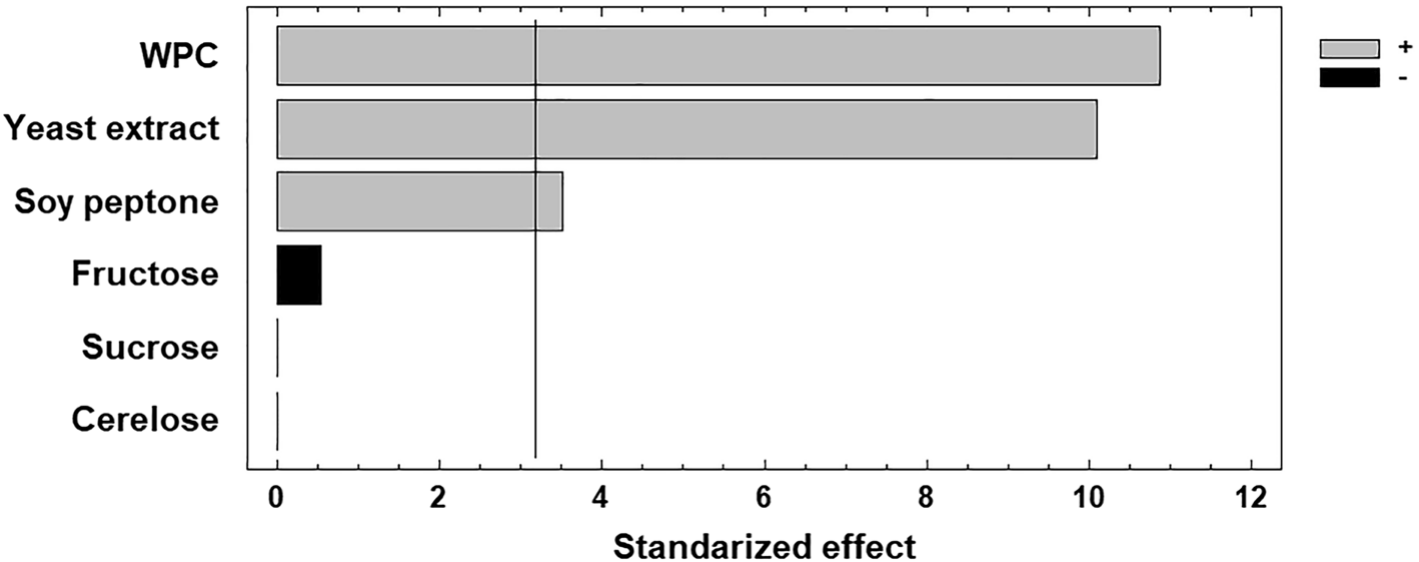
Pareto chart of the effects of six medium components on the biomass production of *L. sakei* ACU-2. The standardized effects of the variables are represented as a single column. The vertical line through the column indicates whether the variables are statistically significant. (■) Positive effect, (■) Negative effect.

Standardized effects of variables on the growth of *L. sakei* ACU-2 are shown in the Pareto chart (Fig. 2). The three studied nitrogen sources exerted significant and positive effects on the response, while carbon sources did not influence biomass production. Consequently, an increase in nitrogen source concentration, from − 1 to + 1, stimulated the growth of the studied strain, as evidenced in runs 9 and 6 (Table 1).

Complex nitrogen sources are crucial in the overproduction of LAB, since these microorganisms are unable to grow in simple culture mediums. Different sources contain different peptides and, depending on the strain, the response to each source will also be different due to specificity of the bacterial enzymes involved^37^. In this context, finding a suitable organic nitrogen source that meets the demanding nutritional requirements of LAB is a very important step in the process of optimizing culture media.

Yeast extract has been extensively reported to enhance biomass production of several species of LAB and its effectiveness is usually attributed to its rich content in nitrogen, carbohydrates, vitamin B, purine and pyrimidine^27,38^. Despite its excellent performance in lactobacilli cultivation, its high cost makes it necessary to look for complementary sources to reduce the concentration of yeast extract added and minimize costs. Soy peptone is usually presented as a safer and cheaper alternative to animal derived sources and has been studied as a replacement in conventional culture media^39^. This vegetable source is a papain digest from soybean meal, which contains carbohydrates, proteins and vitamins; consequently, it is an excellent substrate for fastidious microorganisms, such as LAB, and is commonly found in optimized mediums^4,40,41^. When it comes to WPC, although its contribution to biomass production of microorganisms has been reported^8,42^, its use in culture mediums is not as widespread as that of whey, which is frequently used as a carbon source. Nevertheless, WPC results in an adequate source of nitrogen, given its protein concentration range from 35 to 90% (w/w) and its lower level of fats, in comparison to other forms of whey^43^. Furthermore, WPC has a high content of some amino acids reported as essential for *L. sakei*, such as leucine, isoleucine and valine^44^, which would support the idea of being an appropriate source for the growth of this microorganism.

Analysis of variance of the regression model was significant (*p* < 0.0001) for biomass production of *L. sakei* ACU-2. The adjusted model describing the effect of the significant factors on the response is presented in the following equation:

8 $$Y = 0.47 + 0.06X_{{1}} + 0.04X_{{2}} + 0.02X_{{3}}$$

The determination coefficient (R^2^) was 0.9531, implying that 95.31% of the variation in the response was explained by the model. Besides, the values of adj-R^2^ (0.9414) and pred-R^2^ (0.9151) were in reasonable agreement since the difference between them was smaller than 0.2^27^. Furthermore, the lack of fit test (*p* = 0.89) confirmed adequacy of the model for describing the data. Hence, the adjusted model demonstrated its suitability for predicting the response, as it was verified with the proximity between experimental and predicted values (Table 1).

Although carbon source did not show a significant influence on the growth of *L. sakei* ACU-2, its presence in a culture medium for anaerobic bacteria is indispensable^3^. Therefore, a fixed concentration of 30 g/L of cerelose was incorporated into the basal medium; given it is the cheapest sugar source among the three that were tested.

On the other hand, since the three nitrogen sources assayed significantly influenced microorganism biomass production, a further optimization step was carried out to establish the optimal combination of nitrogen source to be added to the medium in order to obtain a higher cell density.

### Mixture design

In search of the best combination of nitrogen source that promotes the growth of *L. sakei* ACU-2, a simplex centroid mixture design was implemented. The total amount of mixture was set at 30 g/L, since it is crucial to achieve a balance in the concentrations of complex nitrogen sources. These substrates, especially YE and SP, not only contain nitrogen, but are also sources of carbon, minerals, and vitamins. Therefore, higher concentrations can result in growth limitation^25,45^, in addition to raising the cost of the medium.

Dry cell mass results obtained from the mixture design are presented in Table 2. The highest value for the response was obtained in run 7, which represents the ternary mixture of the three components in equal proportion. Conversely, runs 2 and 9—being WPC solely nitrogen source—showed lower biomass production. However, the runs that consisted of binary combinations of WPC with other two sources (runs 4, 6 and their replicates) exhibited higher response values, supporting the results previously mentioned.

ANOVA of the regression models for biomass production of *L. sakei* ACU-2 is shown in Table 3. According to the *p* value, the quadratic model was the most complex model to significantly fit the data (*p* < 0.0001). Moreover, the R^2^ value close to 1 (0.9931) implied that 99.31% of the variation in the response was explained by the model, and the values of adj-R^2^ (0.9888) and pred-R^2^ (0.9813) were in reasonable agreement since the difference between them was smaller than 0.2. These criterions demonstrated that the quadratic model displayed high predictive strength and good fitness; hence, it was the most appropriate model to describe the influence of the variables on biomass production of *L. sakei* ACU-2. Equation of the selected model was as follows:

9 $$Y = 1.43X_{1} + 0.01X_{{2}} + 1.08X_{{3}} + 3.46X_{{1}} X_{{2}} + 0.95X_{{1}} X_{{3}} + 2.76X_{{2}} X_{{3}}$$

**Table 3:** ANOVA of the regression models for biomass production of *L. sakei* ACU-2.

Model	SS	df	R^2^	R^2^ adj	R^2^ pred	*p* value
Lineal	2.00	2	51.91	43.17	16.65	0.0178
Quadratic	1.83	3	99.31	98.88	98.13	< 1 × 10^−4^
Special cubic	0.01	1	99.59	99.23	98.35	0.0667
Error	0.02	7				
Total	24.40	14				

The different values of R^2^ are expressed as %

*p* < 0.05 is significant.

The predictive strength of the quadratic model was further supported by the proximity between experimental and predicted values of dry cell mass, exhibited in Table 2. Additionally, the fitness of the model was verified through the lack of fit, which resulted insignificant (*p* = 0.07). Hence, it proved to be adequate to predict the studied response.

Statistical analysis of the regression coefficients in Eq. (9) revealed that all of the interactions resulted significant (*p* < 0.05) (Table 4), and both the individual components and their interactions had positive effect on the growth of the strain. Furthermore, the combinations of WPC-YE and WPC-SP presented higher coefficients than the sum of the single component coefficients, conferring a synergistic effect to these interactions. On the other hand, even though the interaction between YE and SP also resulted significant, it had a smaller positive effect on the response than that of the individual components.

**Table 4:** ANOVA results of the quadratic model for biomass production of *L. sakei* ACU-2.

Parameter	Coefficient	Standard error	t-value	*p* value
*X*_1_	1.43	0.04		
*X*_2_	0.01	0.04		
*X*_3_	1.08	0.04		
*X*_1_*X*_2_	3.46	0.19	18.52	< 1 × 10^−4^
*X*_1_*X*_3_	0.95	0.19	5.07	0.001
*X*_2_*X*_3_	2.76	0.19	14.77	< 1 × 10^−4^

*X*_1_ Yeast extract; *X*_2_ WPC; *X*_3_ Soy peptone.

*p* < 0.05 is significant.

**Figure 3:**
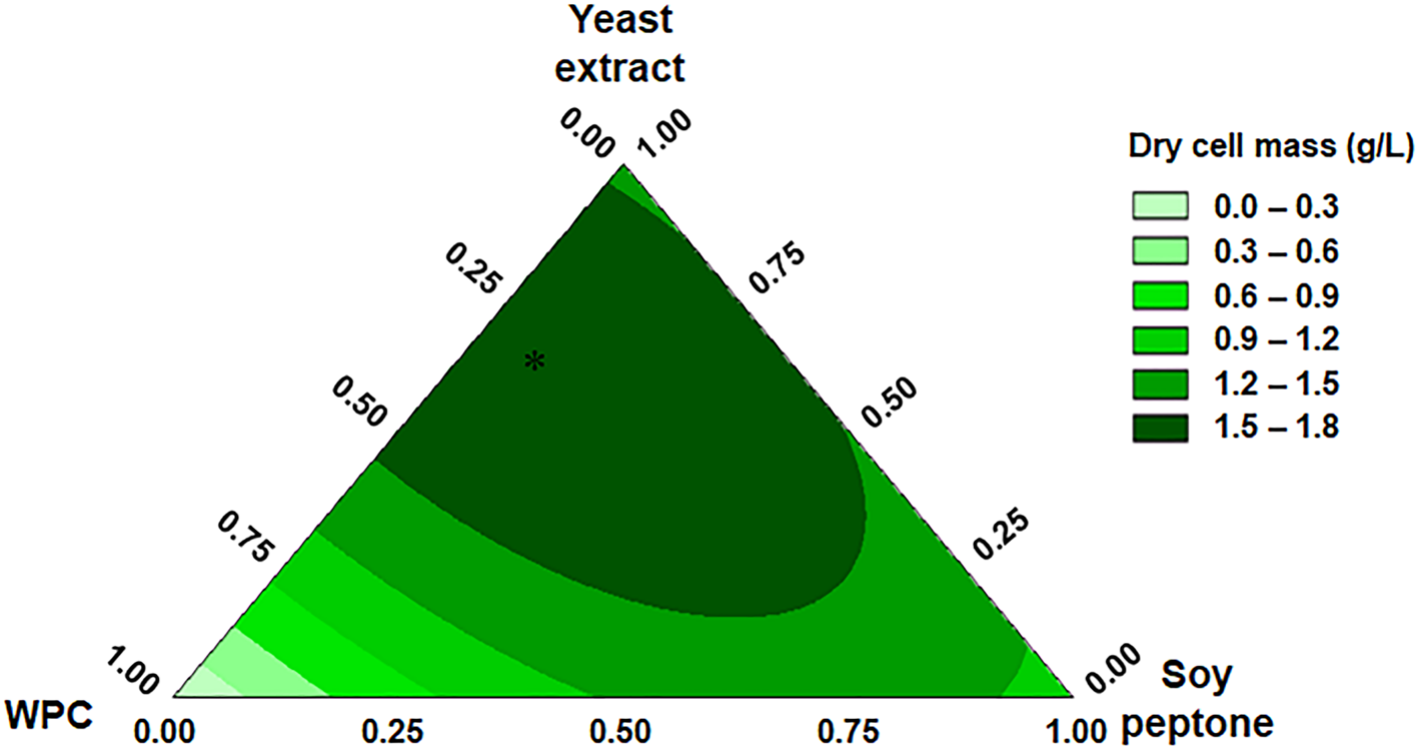
Mixture contour plots for biomass production of *L. sakei* ACU-2 showing the effect of the nitrogen sources. (*) Optimum estimated mixture.

Variations in biomass production obtained from the different mixtures of WPC, YE and SP, are depicted in a two-dimensional contour plot (Fig. 3). In a contour plot, all the points with the same response are connected through contour lines of constant responses, providing a two-dimensional view of the experimental space^46^. The presence of curvature in the plot is explained by the nonlinear blending between the component pairs^47^, since the interaction WPC-YE and WPC-SP have a synergistic effect on the response. Figure 3 also shows that the lowest values of dry cell mass were obtained at the vertices, where WPC and SP were individually represented. The zone of maximum response is located towards the left side of the triangle, having YE and WPC as the vertices, and the optimum estimated mixture consisted of 19.46 g/L of YE, 8.28 g/L of WPC and 2.26 g/L of SP.

The addition of a mixture comprising three nitrogenous sources to the culture medium is justified since each one of them supplies the studied strain with different peptides and amino acids, in order to fulfill its nutritional requirements. In fact, many LAB strains have been reported to be prone to more than one nitrogen source in culture mediums. Choi et al.^9^ developed an optimized medium containing 30.27 g/L YE and 39.43 g/L SP which provided a *L. plantarum* 200655 biomass of 3.845 g/L, 1.58-fold higher than the obtained with the un-optimized medium (2.429 g/L). Yoo et al.^48^ designed a culture medium including 19.7 and 2.13 g/L YE and SP, respectively, and reached a number of viable cells of *L. plantarum* JNU2116 > 10^9^. By means of RSM, Gao et al.^49^ estimated the composition of an economic medium which contained 14.4 g/L peptone, 0.5 g/L urea and 7.3 g/L YE, as the main nitrogen sources for the growth of *L. fermentum*. Fung et al.^50^ achieved maximum growth of *L. acidophilus* FTCC 0291 in a soy whey medium supplemented with a combination of 7.25% (w/v) meat extract, 4.7% (w/v) vegetable extract and 6.85% (w/v) peptone. As *L. sakei* is one of the LAB species with more fastidious nutritional requirements^51^, it is expected that its development requires a wide variety of nutrients. Additionally, the use of the nitrogenous combination enables the replacement of other costly substrates present in the traditional MRS culture medium, such as beef extract, comprising a food-grade mixture.

**Figure 4:**
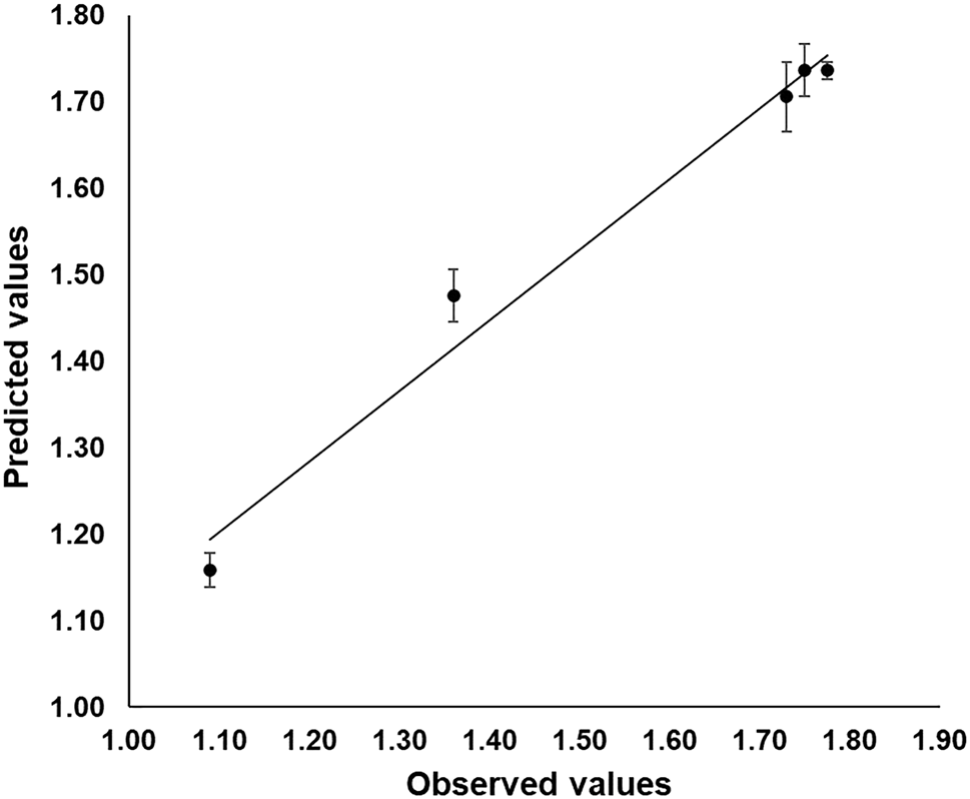
Fitted line plot between the observed and predicted values of dry cell mass in the validation mixtures.

To assess the quality of the predictions of a model, external validation conducted in a different set of conditions is necessary^52^. Consequently, five extra assays consisting of different ternary mixtures were performed, including the optimum estimated mixture. Figure 4 represents the observed and predicted values of dry cell mass obtained with the new mixtures. The R^2^ of 0.9783 suggested a good concordance between the experimental and predicted values.

Model performance indices: bias factor (*BF*), accuracy factor (*AF*), and average mean deviation *E* (%), were also calculated. *BF* assesses the deviations of the observed values from the equivalence line. Accordingly, the *BF* value of 1.01 obtained in this validation indicated that the model had almost no bias and the predictions made were in agreement with the observations. However, since over and under-estimations tends to cancel out, the *BF* value does not indicate the average accuracy of the estimates^53^. On the other hand, *AF* provides information about the accuracy of the estimations and its value is reported to increase by 0.10–0.15 units with each predictive variable in the model^22^. The current model included three variables, thus an *AF* ranging from 1.30 to 1.45 was to be expected. Therefore, the obtained value of 1.04 verified the high agreement between experimental and predicted results. In addition, the error of fit of the data given by *E* (%) resulted in 1%, suggesting that the predictive performance of the model was satisfactory. These results confirmed the validity of the quadratic model for predicting the biomass production by *L. sakei* ACU-2.

Mixture experiments have been successfully applied to culture medium optimization for different purposes, such as screening ingredients^54^, enhancement of exopolysaccharides production^55^, and improvement of functional properties and bacterial growth^46,56^. In this study it proved to be a viable alternative to RSM, as part of a DOE sequence, giving the chance to define the proportions of the mixture components that maximized biomass production when a restriction is imposed.

Thereby, after the optimization process, culture medium composition was defined as follows: 30 g/L cerelose, 19.46 g/L yeast extract, 8.28 g/L WPC, 2.26 g/L soy peptone, 5 g/L sodium acetate, 0.2 g/L magnesium sulfate, 0.05 g/L manganese sulfate, 1 g/L Tween 80.

### Bioreactor cultivation study

Growth performance of *L. sakei* ACU-2 was assessed in a reactor containing the optimum formulated medium, under fixed conditions of pH and temperature (6.5 and 30 °C, respectively). Under the same conditions, this medium performance was compared to MRS culture medium. After 24 h of fermentation, biomass production of the studied strain in the optimized medium was significantly higher than that in MRS broth (*p* < 0.0001). Final dry cell mass concentration obtained with the optimized formulation was of 2.86 ± 0.04 g/L while in MRS was of 1.63 ± 0.06 g/L. The difference among the values represented an enhancement of 75.5% of biomass production by *L. sakei* ACU-2 when fermentation was conducted in the optimized medium.

According to our esS1timations, 1 L of the new medium would cost $3.24, while MRS culture medium cost ranges between $8.51 and $23.51 per liter (depending on the manufacturer) (Supplementary Table S1). Hence, a cost medium reduction of 62–86% could be achieved. This new fermentation medium would be effective and economically viable for the growth of *L. sakei* ACU-2, which is essential for industrial purposes since 30–40% of the production costs is assigned to the culture medium^56^.

## Conclusions

The optimization sequence performed in this study proved to be appropriate to design a low-cost food grade culture medium for biomass production of *L. sakei* ACU-2.

Replacing the ingredients of the MRS broth with industrial-grade substrates and by-products not only made it possible to achieve higher biomass values, but also considerably reduced the overall cost. In addition, the accomplished balance between alternative and chemical defined components in the optimized formulation will lessen the yield variation among batches in future scale-ups fermentation processes.

On the other hand, the intermediate operation steps, namely those aimed to remove non-food grade components, between fermentation and any preservation technique (such as spray drying) could be avoided since the composition of the optimized medium has food grade status. In conclusion, the whole process—biomass production and drying—could be considered as cost-efficient, giving a superlative advantage. Further studies are needed, nonetheless, in order to evaluate the survival of the strain after the drying process.

## Supplementary Information

Supplementary Information.

## Data Availability

The datasets generated and analyzed during the current study are available from the corresponding author upon reasonable request.
