# Supplementary material for: Alternative culture medium design for biomass production of autochthonous meat starter Latilactobacillus sakei sp. sakei ACU-2
Source: Sci Rep. 2023 Mar 30;13:5205. doi: 10.1038/s41598-023-29502-2 (PMC10063532; doi:10.1038/s41598-023-29502-2)
Supplement: Supplementary file 1 — Supplementary Information. [file 41598_2023_29502_MOESM1_ESM.pdf]

Alternative culture medium design for biomass production of autochthonous meat starter *Latilactobacillus sakei* sp. *sakei* ACU-2

Nadia Galante; Noelia Palavecino Prpich; Carmen Campos; María Elisa Cayré; Marcela Castro

Supplementary Material

Table S1. Cost comparison between the optimized medium and commercial MRS medium.

Commercial MRS	Contents (g/L)	Price per liter ($)	Optimized Medium Composition	Contents (g/L)	Price per liter ($)
Brand 1	55.20	23.51	Cerelose	30.00	0.11
Brand 2	55.00	12.90	Yeast extract	19.46	1.92
Brand 3	55.30	8.97	Soy peptone	2.26	0.54
Brand 4	55.25	8.51	WPC	8.28	0.22
			Tween 80	1.00	0.09
			Sodium acetate	5.00	0.34
			$MgSO_4$	0.20	0.01
			$MnSO_4$	0.05	0.01
			**Total price ($)**		**3.24**

The price was acquired from the market in November 2022, Argentina.
